# Application of FLOW 800 in extracranial-to-intracranial bypass surgery for moyamoya disease

**DOI:** 10.3171/2021.10.FOCVID21191

**Published:** 2022-01-01

**Authors:** Benjamin Yim, Andrew J. Gauden, Gary K. Steinberg

**Affiliations:** Department of Neurosurgery, Stanford University School of Medicine, Stanford, California

**Keywords:** moyamoya disease, FLOW 800, bypass, indocyanine green, ICG, videoangiography, extracranial-to-intracranial

## Abstract

The surgical treatment of moyamoya disease is heavily reliant upon a real-time understanding of cerebral hemodynamics. The application of FLOW 800 allows the surgeon to semiquantify the degree of perfusion to the cerebral cortex following extracranial-to-intracranial (EC-IC) bypass surgery. The authors present three illustrative cases demonstrating common intraoperative findings prior to and following anastomosis using FLOW 800. All patients were diagnosed by catheter angiogram with moyamoya disease and noninvasive imaging demonstrating hemispheric hypoperfusion. Superficial temporal artery (STA)–to–middle cerebral artery (MCA or M4) bypasses were performed to augment intracranial perfusion. The patients tolerated the procedures well and were discharged without event in stable neurological condition.

The video can be found here: https://stream.cadmore.media/r10.3171/2021.10.FOCVID21191

## Transcript

This is a video demonstrating the application of FLOW 800 in extracranial-to-intracranial bypass surgery for moyamoya disease.^1–5^

### 0:29 FLOW 800.

The FLOW 800 software designed by Zeiss utilizes indocyanine green–based videoangiography performed at a region of interest through a semiquantitative analysis based on time to half-maximum intensity and florescence intensity. The results are viewed in two-dimensional color maps in the form of delay, intensity, and speed. For simplicity, we will only focus on the delay maps in this video.

### 0:55 Case Presentation.

Our case demonstration begins with an 18-year-old male with Down syndrome presenting with recurrent episodes of left facial droop, left arm numbness, and dysarthria lasting minutes with complete resolution. On clinical exam, he demonstrates moderate cognitive impairment but otherwise neurologically intact.

### 1:15 Imaging.

Preoperative digital subtraction angiography demonstrates an occlusion of the right internal carotid artery with reconstitution of the right M1 segment via moyamoya collaterals and delayed right hemisphere perfusion. An extracranial-to-intracranial bypass is recommended, and the patient and family were agreeable.

### 1:37 Case 1: Surgical Procedure.

After mapping the parietal division of the superficial temporal artery using a Doppler, an incision is made and the superficial temporal artery is skeletonized prior to performing a frontotemporal craniotomy. The dura is opened in a stellate fashion and the STA is positioned along the posterior margin. A suitable M4 recipient site is identified and ICG videoangiography is performed for baseline FLOW 800 data. As is typical in moyamoya patients, there is hyperemia along the cortical surface. The recipient vessel measures 1.2 mm in diameter, and the distal STA measures to be of roughly equivalent size. A flowmeter is used to determine the cut flow velocity of the STA. The donor vessel is then temporarily occluded and flushed with heparinized saline. Temporary clips are used to occlude the recipient vessel and an ellipsoid arteriotomy is performed. The site is flushed with heparinized saline, followed by indigo carmine dye. Once the heel and toe are sutured, an end-to-side anastomosis is performed using 10-0 sutures in an interrupted fashion. Following completion of the anastomosis, the temporary clips are removed, and hemostasis is achieved.

### 3:04 Overview.

The total occlusion time for this case was 16 minutes. Postanastomosis ICG videoangiography is then performed and the FLOW 800 data is processed.

### 3:23 FLOW 800 Map 1.

When comparing the pre- and postanastomosis color maps, the previously seen blue and green that dominated the preanastomosis map was replaced with more yellow-orange hues consistent with decreased delay and increased perfusion along the cortical surface. The flowmeter found prebypass maximal M4 flow to be retrograde at 5.04 ml/min. Following bypass, proximal M4 had retrograde flow of 5.76 ml/min and antegrade flow of 28.4 ml/min along the distal M4.

### 3:54 Postoperative Angiogram.

Postoperative angiogram at 6 months demonstrates patency of the bypass with supply to 75% of the middle cerebral artery distribution. The patient remained neurologically at his baseline without event at 1 year postoperatively.

### 4:14 Case 2: Prebypass Competing Flows.

The next case demonstrates the concept of prebypass competing flows. This patient also underwent an EC-IC bypass for symptomatic moyamoya disease; however, the prebypass ICG videoangiography demonstrates retrograde filling of the distal MCA by collaterals competing with the antegrade flow from stenosed vessels proximally. Following an end-to-side STA-MCA bypass, flow appears to be dominated by the bypass.

### 4:51 FLOW 800 Map 2.

The FLOW 800 color map demonstrates improved perfusion diffusely along the cortical surface with diminished delay following the bypass. Flowmeter measurements demonstrate preanastomosis M4 flow to be 1.4 ml/min. Postbypass, proximal M4 appears to fill retrograde at 12.3 ml/min and distal M4 fills anterograde at 37.8 ml/min.

### 5:20 Case 3: Postbypass Competing Flows.

In contrast, this last case demonstrates the development of competing flows following bypass. Similar moyamoya patient undergoing an STA-MCA bypass had prebypass ICG videoangiography demonstrating delayed anterograde filling of the M4 branch. Postbypass, ICG is visualized filling the proximal M4 antegrade until reaching the anastomosis site, with only trickling into the distal M4 branches. During the end of the injection, ICG is visualized in the STA and dominating the flow into the distal M4 in an anterograde fashion, with minimal flow into the proximal M4 demonstrating competing flow between the graft and the proximal M4.

### 6:12 FLOW 800 Map 3.

Nevertheless, FLOW 800 color maps demonstrate increased perfusion of the cortical surface.

### 6:20 Pearls for Effective FLOW 800.

In regard to the keys in obtaining accurate FLOW 800 data, the injections must be performed through the same intravenous access point under comparable hemodynamic pressures. There must be close communication with the anesthesia team to ensure that the rate and volume of ICG and subsequent flush are reproducible. Finally, a vital tool is the PositionMemory feature of the robotic visualization system for microscopic accuracy between maps. Overall, FLOW 800 provides the surgeon with real-time information regarding cortical perfusion previously not available.

## Disclosures

Dr. Steinberg reports being a consultant for Peter Lazic US, NeuroSave, SanBio, Audaxion Therapeutics, Zeiss, and Surgical Theater.

## Author Contributions

Primary surgeon: Steinberg. Editing and drafting the video and abstract: all authors. Critically revising the work: all authors. Reviewed submitted version of the work: all authors. Approved the final version of the work on behalf of all authors: Steinberg. Supervision: Steinberg.
